# *rac*-Hy­droxy­isovaleric acid

**DOI:** 10.1107/S2414314623010933

**Published:** 2024-01-05

**Authors:** Lukhanyo Dasi, Eric Cyriel Hosten, Richard Betz

**Affiliations:** a Nelson Mandela University, Summerstrand Campus, Department of Chemistry, University Way, Summerstrand, PO Box 77000, Port Elizabeth, 6031, South Africa; Goethe-Universität Frankfurt, Germany

**Keywords:** crystal structure, hydrogen bonding, hy­droxy­carb­oxy­lic acids

## Abstract

The title compound (systematic name: *rac*-2-hydroxy-3-methylbutanoic acid), C_5_H_10_O_3_, is the constitutional isomer of α-hy­droxy­butanoic acid. In the crystal, hydrogen bonds involving the alcoholic hydroxyl group give rise to centrosymmetric dimers that are extended to sheets perpendicular to the crystallographic *c* axis.

## Abstract 2

The title compound is the constitutional isomer of α-hy­droxy­butanoic acid. In the crystal, hydrogen bonds involving the alcoholic hydroxyl group give rise to centrosymmetric dimers that are extended to sheets perpendicular to the crystallographic *c* axis.

## Graphical abstract

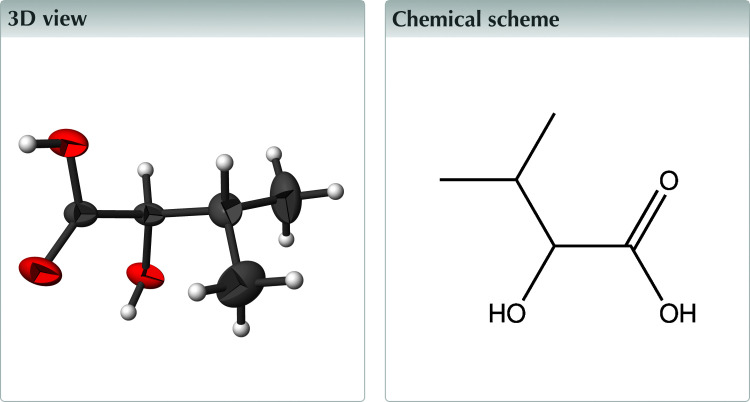

## Structure description

The Krebs Cycle – also known as Citric Acid Cycle – is at the centre of metabolic processes in aerobic organisms. It involves a number of hy­droxy­carb­oxy­lic acids that constitute intriguing chelating ligands for a variety of transition metals of pharmaceutical inter­est (McMurry, 2008 ▸). These potential ligands classify as chelate ligands, which have found widespread use in coordination chemistry due to the increased stability of coordination compounds they can form in comparison to monodentate ligands (Gade, 1998 ▸). Hy­droxy­carb­oxy­lic acids are a particularly inter­esting class of ligands as they offer two functional groups that, depending on the experimental conditions, can either act as fully neutral, fully anionic or mixed neutral-anionic donors. Upon varying the substitution pattern on the hydro­carbon backbone, the acidity of the respective hydroxyl groups can be fine-tuned over a wide range and they may, thus, serve as probes for establishing the rules in which pK_a_ range coordination to various central atoms can be observed. Furthermore, the steric pretence of potential substituents may give rise to unique coordination and bonding patterns. Given the multidentate nature of hy­droxy­carb­oxy­lic acids encountered in the Krebs Cycle it appears prudent to investigate simpler ‘cut outs’ with a more limited number of donor sites to avoid complexer mixtures of reaction products in envisioned synthesis procedures, thus prompting the diffraction study of the title compound to allow for comparisons of metrical parameters of the free ligand and the ligand in envisioned coordination compounds. The present study falls into the ambit of our continued inter­est into structural aspects of *alpha*-hy­droxy­carb­oxy­lic acids such as 1-hy­droxy­cyclo­propane­carb­oxy­lic acid (Betz & Klüfers, 2007*a* ▸), 1-hy­droxy­cyclo­butane­carb­oxy­lic acid (Betz & Klüfers, 2007*b* ▸), 1-hy­droxy­cyclo­penta­necarb­oxy­lic acid (Betz & Klüfers, 2007*c* ▸) or *tert*-butyl­glycolic acid (Betz *et al.*, 2007 ▸). Furthermore, geometrical data for glycolic acid (Ellison *et al.*, 1971 ▸; Pijper, 1971 ▸) and l-lactic acid (Schouten *et al.*, 1994 ▸; Yang *et al.*, 2021 ▸) is apparent in the literature while, to the best of our knowledge, none of the various hy­droxy-*n*-butanoic acids have been subjected to diffraction studies. Only one report provides the crystal and mol­ecular structure for *gamma*-hy­droxy­butanoic acid as a solvent mol­ecule in a barium-supported tetra­phenyl­imidodiphosphinato compound (Morales-Juarez *et al.*, 2005 ▸).

**Figure 1:**
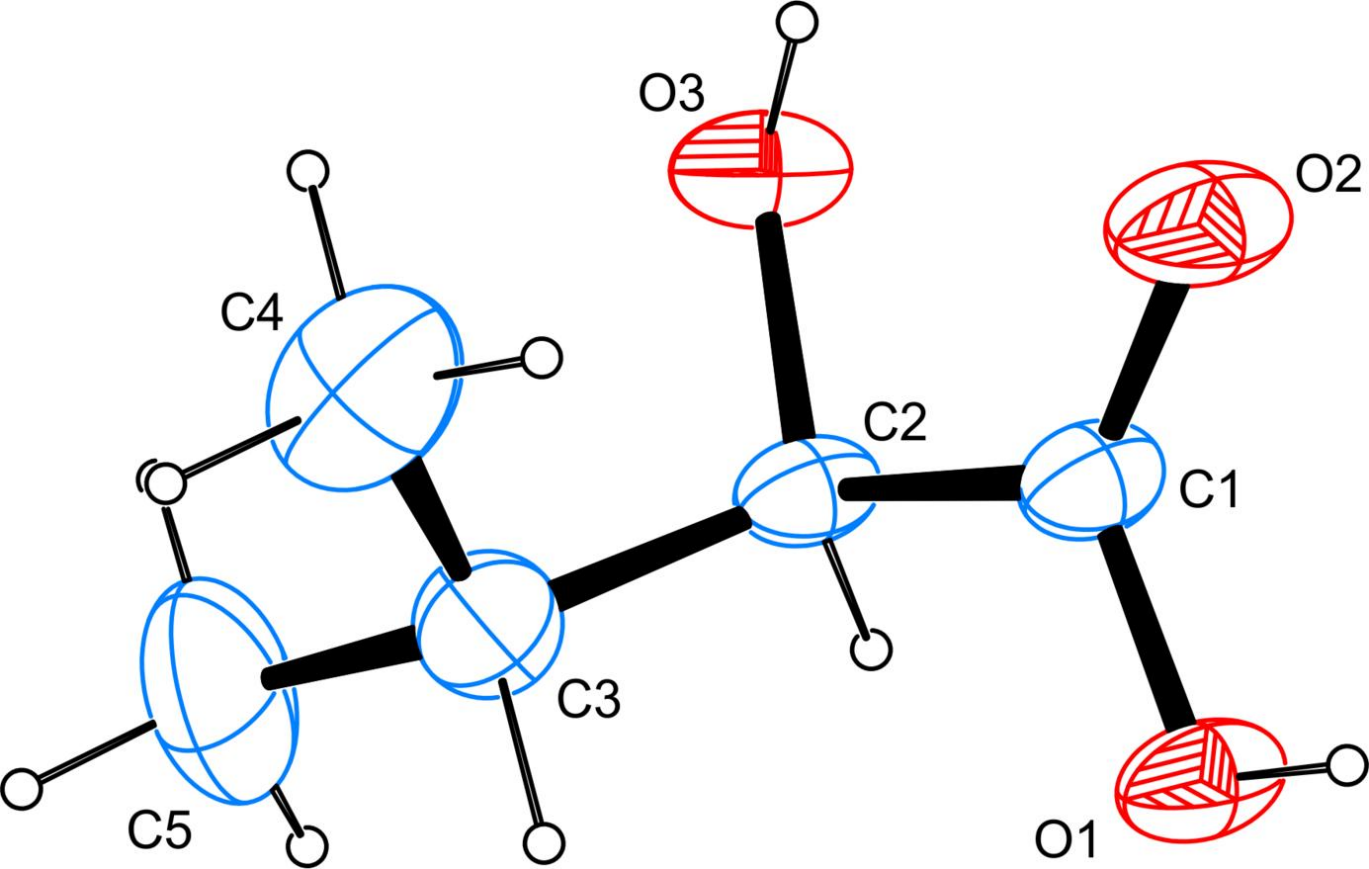
The mol­ecular structure of the title compound, with atom labels and anisotropic displacement ellipsoids (drawn at 50% probability level).

The asymmetric unit of the title compound is shown in Fig. 1 ▸ and contains one complete mol­ecule. C—O bond lengths are found to be 1.4175 (11) Å for the alcoholic hydroxyl group and 1.2064 (12) and 1.3143 (11) Å for the carb­oxy­lic acid group and, thus, lie in the normal range reported for other hy­droxy­carb­oxy­lic acids whose metrical parameters have been deposited with the Cambridge Structural Database (Groom *et al.*, 2016 ▸). The alcoholic hy­droxy group adopts a staggered conformation relative to the two terminal methyl groups with the relevant C—C—C—O torsional angles measuring −58.32 (11) and 66.60 (12)°.

**Figure 2:**
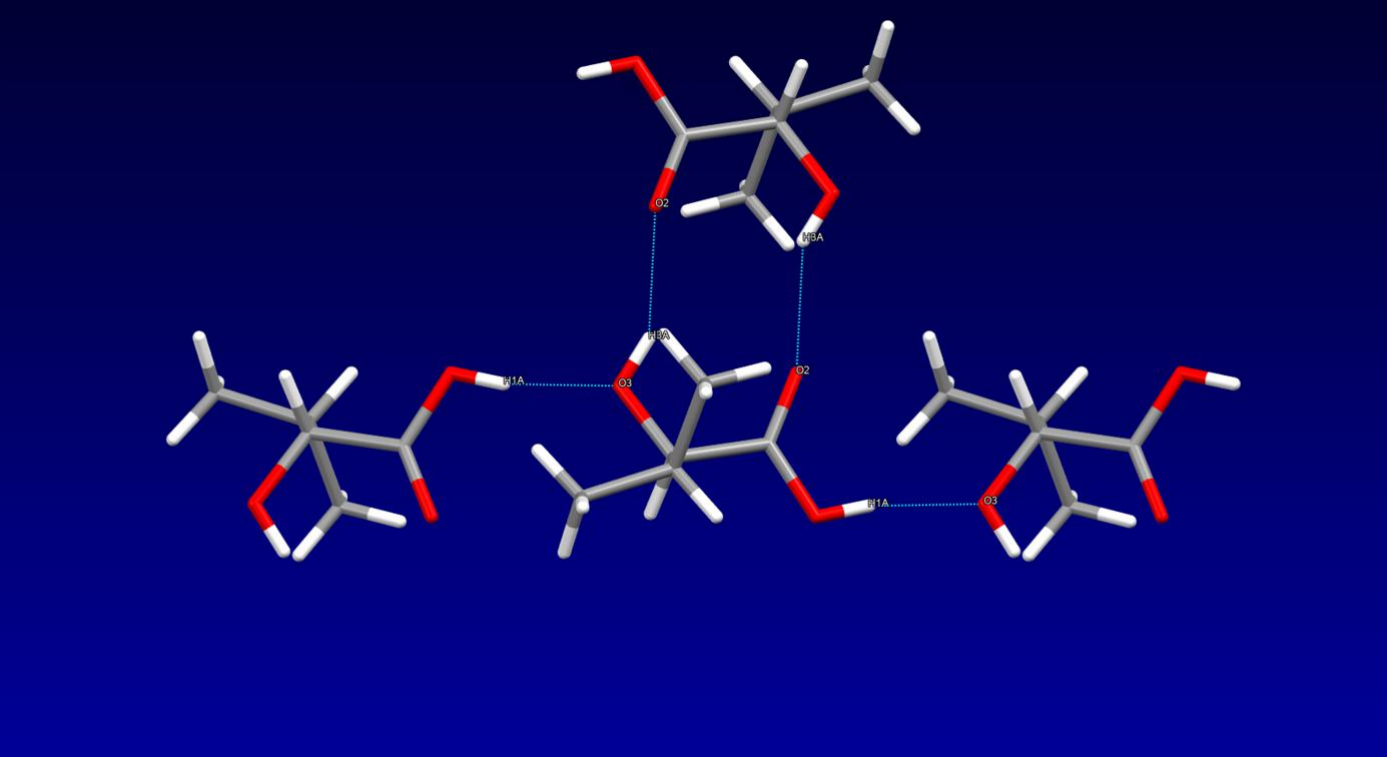
Inter­molecular hydrogen bonds (dotted blue lines), viewed along [001].

In the crystal, classical hydrogen bonds of the O—H⋯O type (Table 1 ▸) are apparent that involve all hydroxyl groups as donors and the oxygen atom of the alcoholic hydroxyl group and the carbonyl oxygen atom as acceptors. The hydrogen bonds supported by the alcoholic hydroxyl group as donor and the carbonyl oxygen atom as acceptor connect the individual mol­ecules into centrosymmetric dimers, which are further extended to sheets perpendicular to the crystallographic *c* axis by means of the carb­oxy­lic acid’s hydroxyl group as donor and the alcoholic hydroxyl group’s oxygen atom as acceptor. In terms of graph-set analysis (Etter *et al.*, 1990 ▸; Bernstein *et al.*, 1995 ▸), the descriptor for these hydrogen bonds is 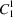 (5) 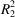 (10) on the unary level. A depiction of the hydrogen-bonding pattern is shown in Fig. 2 ▸.

## Synthesis and crystallization

The compound was obtained commercially (Fluka). Crystals suitable for the diffraction studies were taken directly from the provided material.

## Refinement

Crystal data, data collection and structure refinement details are summarized in Table 2 ▸.

## Supplementary Material

Crystal structure: contains datablock(s) I, global. DOI: 10.1107/S2414314623010933/bt4145sup1.cif

Structure factors: contains datablock(s) I. DOI: 10.1107/S2414314623010933/bt4145Isup2.hkl

Supporting information file. DOI: 10.1107/S2414314623010933/bt4145Isup3.cml

CCDC reference: 2320961

Additional supporting information:  crystallographic information; 3D view; checkCIF report

## Figures and Tables

**Table 1:** Hydrogen-bond geometry (Å, °)

*D*—H⋯*A*	*D*—H	H⋯*A*	*D*⋯*A*	*D*—H⋯*A*
O1—H1*A*⋯O3^i^	0.84	1.78	2.6143 (9)	169
O3—H3*A*⋯O2	0.84	2.32	2.7069 (10)	108
O3—H3*A*⋯O2^ii^	0.84	2.00	2.7597 (11)	150

Symmetry codes: (i) 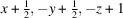 ; (ii) 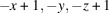 .

**Table 2:** Experimental details

Crystal data	
Chemical formula	C_5_H_10_O_3_
*M*_r_	118.13
Crystal system, space group	Orthorhombic, *P* *b* *c* *a*
Temperature (K)	200
*a*, *b*, *c* (Å)	10.9589 (4), 9.3280 (4), 12.7255 (6)
*V* (Å^3^)	1300.86 (10)
*Z*	8
Radiation type	Mo *K*α
μ (mm^−1^)	0.10
Crystal size (mm)	0.60 × 0.51 × 0.35

Data collection	
Diffractometer	Bruker (2010 ▸) APEXII CCD
Absorption correction	Numerical (*SADABS*, Krause *et al.*, 2015 ▸)
*T*_min_, *T*_max_	0.928, 0.990
No. of measured, independent and observed [*I* > 2σ(*I*)] reflections	10603, 1620, 1366
*R*_int_	0.017
(sin θ/λ)_max_ (Å^−1^)	0.669

Refinement	
*R*[*F* ^2^ > 2σ(*F* ^2^)], *wR*(*F* ^2^), *S*	0.035, 0.107, 1.05
No. of reflections	1620
No. of parameters	79
H-atom treatment	H-atom parameters constrained
Δρ_max_, Δρ_min_ (e Å^−3^)	0.35, −0.15

Computer programs: *APEX2* and *SAINT* (Bruker, 2010 ▸), *SHELXS97*, *SHELXL97* and *SHELXTL* (Sheldrick, 2008 ▸)*SHELXTL* (Sheldrick, 2008 ▸), *PLATON* (Spek, 2020 ▸) and *Mercury* (Macrae *et al.*, 2020 ▸).

## full crystallographic data

### Crystal data

**Table:**

C_5_H_10_O_3_	*D*_x_ = 1.206 Mg m^−^^3^
*M**_r_* = 118.13	Mo *K*α radiation, λ = 0.71073 Å
Orthorhombic, *P**b**c**a*	Cell parameters from 4732 reflections
*a* = 10.9589 (4) Å	θ = 3.2–28.1°
*b* = 9.3280 (4) Å	µ = 0.10 mm^−^^1^
*c* = 12.7255 (6) Å	*T* = 200 K
*V* = 1300.86 (10) Å^3^	Blocks, colourless
*Z* = 8	0.60 × 0.51 × 0.35 mm
*F*(000) = 512	

### Data collection

**Table:**

Bruker (2010) APEXII CCD diffractometer	1620 independent reflections
Radiation source: sealed tube	1366 reflections with *I* > 2σ(*I*)
Graphite monochromator	*R*_int_ = 0.017
φ and ω scans	θ_max_ = 28.4°, θ_min_ = 3.2°
Absorption correction: numerical (SADABS, Krause *et al.*, 2015)	*h* = −11→14
*T*_min_ = 0.928, *T*_max_ = 0.990	*k* = −12→12
10603 measured reflections	*l* = −16→16

### Refinement

**Table:**

Refinement on *F*^2^	Primary atom site location: structure-invariant direct methods
Least-squares matrix: full	Secondary atom site location: difference Fourier map
*R*[*F*^2^ > 2σ(*F*^2^)] = 0.035	Hydrogen site location: inferred from neighbouring sites
*wR*(*F*^2^) = 0.107	H-atom parameters constrained
*S* = 1.05	*w* = 1/[σ^2^(*F*_o_^2^) + (0.0586*P*)^2^ + 0.2192*P*] where *P* = (*F*_o_^2^ + 2*F*_c_^2^)/3
1620 reflections	(Δ/σ)_max_ < 0.001
79 parameters	Δρ_max_ = 0.35 e Å^−^^3^
0 restraints	Δρ_min_ = −0.15 e Å^−^^3^

### Special details

**Table:**

Geometry. All esds (except the esd in the dihedral angle between two l.s. planes) are estimated using the full covariance matrix. The cell esds are taken into account individually in the estimation of esds in distances, angles and torsion angles; correlations between esds in cell parameters are only used when they are defined by crystal symmetry. An approximate (isotropic) treatment of cell esds is used for estimating esds involving l.s. planes.
Refinement. The carbon-bound H atom of the methine group was placed in a calculated position1 (C–H 1.00 Å) and was included in the refinement in the riding model approximation, with *U*(H) set to 1.2*U*_eq_(C).The H atoms of the methyl groups were allowed to rotate with a fixed angle around the C–C bond to best fit the experimental electron density (HFIX 137 in the *SHELX* program suite (Sheldrick, 2008)), with *U*(H) set to 1.5*U*_eq_(C).The H atoms of the hydroxyl groups were allowed to rotate with a fixed angle around the C–C bond to best fit the experimental electron density (HFIX 147 in the *SHELX* program suite (Sheldrick, 2008)), with *U*(H) set to 1.5*U*_eq_(O).

### Fractional atomic coordinates and isotropic or equivalent isotropic displacement parameters (Å^2^)

**Table:**

	*x*	*y*	*z*	*U*_iso_*/*U*_eq_	
O1	0.62325 (6)	0.36594 (8)	0.47438 (7)	0.0378 (2)	
H1A	0.695518	0.348157	0.492101	0.066 (5)*	
O2	0.59660 (8)	0.13436 (8)	0.51070 (8)	0.0464 (3)	
O3	0.35374 (6)	0.15502 (8)	0.47493 (7)	0.0341 (2)	
H3A	0.394355	0.078645	0.474626	0.049 (4)*	
C1	0.55798 (9)	0.24797 (10)	0.47994 (8)	0.0288 (2)	
C2	0.42861 (8)	0.26994 (9)	0.44097 (8)	0.0266 (2)	
H2	0.396479	0.360413	0.472773	0.032*	
C3	0.42813 (9)	0.28752 (12)	0.32116 (8)	0.0379 (3)	
H3	0.486954	0.365679	0.303082	0.046*	
C4	0.47104 (15)	0.15122 (17)	0.26654 (11)	0.0592 (4)	
H4A	0.472586	0.166730	0.190382	0.089*	
H4B	0.414942	0.072567	0.283046	0.089*	
H4C	0.553206	0.126684	0.291045	0.089*	
C5	0.30193 (13)	0.3343 (2)	0.28387 (12)	0.0647 (4)	
H5A	0.303823	0.351007	0.207865	0.097*	
H5B	0.278457	0.422888	0.319944	0.097*	
H5C	0.242457	0.258894	0.299861	0.097*	

### Atomic displacement parameters (Å^2^)

**Table:**

	*U*^11^	*U*^22^	*U*^33^	*U*^12^	*U*^13^	*U*^23^
O1	0.0214 (3)	0.0242 (4)	0.0678 (5)	−0.0016 (3)	−0.0083 (3)	0.0040 (3)
O2	0.0317 (4)	0.0245 (4)	0.0830 (6)	0.0016 (3)	−0.0136 (4)	0.0091 (4)
O3	0.0187 (3)	0.0242 (4)	0.0593 (5)	0.0019 (2)	0.0065 (3)	0.0076 (3)
C1	0.0218 (4)	0.0216 (4)	0.0431 (5)	0.0010 (3)	−0.0013 (4)	−0.0008 (3)
C2	0.0192 (4)	0.0190 (4)	0.0417 (5)	0.0006 (3)	0.0001 (4)	0.0004 (3)
C3	0.0299 (5)	0.0418 (6)	0.0421 (6)	−0.0069 (4)	−0.0015 (4)	0.0056 (4)
C4	0.0572 (8)	0.0690 (9)	0.0514 (7)	−0.0101 (7)	0.0171 (6)	−0.0167 (6)
C5	0.0441 (7)	0.0949 (12)	0.0550 (7)	0.0007 (7)	−0.0172 (6)	0.0153 (7)

### Geometric parameters (Å, º)

**Table:**

O1—C1	1.3143 (11)	C3—C5	1.5257 (17)
O1—H1A	0.8400	C3—H3	1.0000
O2—C1	1.2064 (12)	C4—H4A	0.9800
O3—C2	1.4175 (11)	C4—H4B	0.9800
O3—H3A	0.8400	C4—H4C	0.9800
C1—C2	1.5159 (13)	C5—H5A	0.9800
C2—C3	1.5334 (14)	C5—H5B	0.9800
C2—H2	1.0000	C5—H5C	0.9800
C3—C4	1.5234 (18)		
			
C1—O1—H1A	109.5	C5—C3—H3	107.7
C2—O3—H3A	109.5	C2—C3—H3	107.7
O2—C1—O1	124.19 (9)	C3—C4—H4A	109.5
O2—C1—C2	123.57 (9)	C3—C4—H4B	109.5
O1—C1—C2	112.22 (8)	H4A—C4—H4B	109.5
O3—C2—C1	109.83 (7)	C3—C4—H4C	109.5
O3—C2—C3	112.46 (8)	H4A—C4—H4C	109.5
C1—C2—C3	110.06 (8)	H4B—C4—H4C	109.5
O3—C2—H2	108.1	C3—C5—H5A	109.5
C1—C2—H2	108.1	C3—C5—H5B	109.5
C3—C2—H2	108.1	H5A—C5—H5B	109.5
C4—C3—C5	112.11 (12)	C3—C5—H5C	109.5
C4—C3—C2	111.31 (10)	H5A—C5—H5C	109.5
C5—C3—C2	110.05 (9)	H5B—C5—H5C	109.5
C4—C3—H3	107.7		
			
O2—C1—C2—O3	17.39 (14)	O3—C2—C3—C4	−58.32 (11)
O1—C1—C2—O3	−163.81 (8)	C1—C2—C3—C4	64.49 (11)
O2—C1—C2—C3	−106.95 (12)	O3—C2—C3—C5	66.60 (12)
O1—C1—C2—C3	71.85 (10)	C1—C2—C3—C5	−170.59 (10)

### Hydrogen-bond geometry (Å, º)

**Table:**

*D*—H···*A*	*D*—H	H···*A*	*D*···*A*	*D*—H···*A*
O1—H1*A*···O3^i^	0.84	1.78	2.6143 (9)	169
O3—H3*A*···O2	0.84	2.32	2.7069 (10)	108
O3—H3*A*···O2^ii^	0.84	2.00	2.7597 (11)	150

Symmetry codes: (i) *x*+1/2, −*y*+1/2, −*z*+1; (ii) −*x*+1, −*y*, −*z*+1.
